# Demonstration of self-truncated ionization injection for GeV electron beams

**DOI:** 10.1038/srep14659

**Published:** 2015-10-01

**Authors:** M. Mirzaie, S. Li, M. Zeng, N. A. M. Hafz, M. Chen, G. Y. Li, Q. J. Zhu, H. Liao, T. Sokollik, F. Liu, Y. Y. Ma, L.M. Chen, Z. M. Sheng, J. Zhang

**Affiliations:** 1 Key Laboratory for Laser Plasmas (MOE) and Department of Physics and Astronomy, Shanghai Jiao Tong University, Shanghai 200240, China; 2 IFSA Collaborative Innovation Center, Shanghai Jiao Tong University, Shanghai 200240, China; 3 College of Science, National University of Defense Technology, Changsha 410073, China; 4 Beijing National Laboratory of Condensed Matter Physics, Institute of Physics, CAS, Beijing 100190, China; 5 SUPA, Department of Physics, University of Strathclyde, Glasgow G4 0NG, UK

## Abstract

Ionization-induced injection mechanism was introduced in 2010 to reduce the laser intensity threshold for controllable electron trapping in laser wakefield accelerators (LWFA). However, usually it generates electron beams with continuous energy spectra. Subsequently, a dual-stage target separating the injection and acceleration processes was regarded as essential to achieve narrow energy-spread electron beams by ionization injection. Recently, we numerically proposed a self-truncation scenario of the ionization injection process based upon overshooting of the laser-focusing in plasma which can reduce the electron injection length down to a few hundred micrometers, leading to accelerated beams with extremely low energy-spread in a single-stage. Here, using 100 TW-class laser pulses we report experimental observations of this injection scenario in centimeter-long plasma leading to the generation of narrow energy-spread GeV electron beams, demonstrating its robustness and scalability. Compared with the self-injection and dual-stage schemes, the self-truncated ionization injection generates higher-quality electron beams at lower intensities and densities, and is therefore promising for practical applications.

Laser wakefield electron acceleration (LWFA) was proposed in 1979 by Tajima and Dawson^1^. In this scheme, an intense focused laser pulse (*I* ≥ 10^18^ W/cm^2^) propagates through underdense plasma and excites in its wake relativistic plasma waves with field amplitudes reaching 100 GV/m, making it a perfect structure for electron acceleration to relativistic energy over extremely short distances. Therefore, LWFA is foreseen as a promising scheme for building compact high-energy accelerators^2^ in the future. It may also be compact drivers for X-ray light sources and free electron lasers^3^,^4^,^5^,^6^. A major advance in LWFA was the experimental demonstration of quasi-mono-energetic (QME) electron beams reported in 2004^7^,^8^,^9^. The generation of QME beams has been attributed to the laser wakefield excitation in the nonlinear bubble regime^10^, triggering self-injection of electrons into the wakefield. Since then a remarkable progress in this research area has been made^11^,^12^,^13^,^14^. However, intensive studies^15^,^16^ have shown that large normalized vector potential 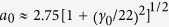 (i.e. high laser intensity) is required for the self-injection (SI) process to take place. Here 
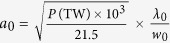
, 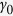
is the relativistic factor of wake phase velocity, *P* is the laser power in units of terawatt (1 TW = 10^12^ W), *λ*_0_ and *w*_0_ are the laser wavelength and focal spot size in units of micrometers (μm), respectively. Therefore, it is necessary to discover ways to lower the laser intensity threshold for electron injection and in the meanwhile to make the injection process more controllable than the self-injection.

Recently, the “ionization-induced electron injection” mechanism^17^,^18^ was proposed to achieve this goal. It utilizes the high ionization-threshold for the K-shell electrons of a high-Z gas (such as nitrogen), which is mixed with the low-Z gas (He) in order to control the initial injection phase of the ionized K-shell electrons. Because a gas mixture target is relatively simple, the ionization injection is more simple to implement compared with other controlled injection schemes such as the optical injection and density down-ramp^19^,^20^. However, electron beams produced by this mechanism usually had large energy-spreads reaching 60−100%^17^,^21^, thus the possible applications of those beams are quite limited. For example, in an attempt^22^ to utilize ionization injection in the interaction of 110 TW laser with 1.2 cm-long gas mixture of 97% He and 3% CO_2_, even though the electron energy was boosted up to ≈1.5 GeV, the energy spectrum was continuous^22^. The fundamental reason for such large energy-spread is the continuous occurrence of ionization injection throughout the whole mixed gas target. Consequently, a dual-stage target with a short injection stage of doped gas was proposed in order to improve the relative energy-spread^23^,^24^. Up to date this scheme^24^ has produced a spectrum of multiple electron bunches up to 800 MeV in energy with an energy-spread of 25%.

Based on particle-in-cell (PIC) simulations^25^ we have shown a self-truncation of the ionization injection (STII) process, where the electron injection occurs only over a distance of few-hundred micrometers at the very front region of the He-N_2_ mixed gas target. The main advantage of the STII is the fact that it is a single-stage scheme which can generate as low as few percent energy-spread electron beams. This is realized by using the so-called unmatched large laser spot size at the beginning, where the laser intensity is high-enough to ionize the K-shell electrons of N_2_, injecting them into the wake formed in the background He plasma. During the laser self-focusing process, the wake wave is transversely narrowed and longitudinally extended. The ionization position of some K-shell electrons moves relatively forward inside the wake such that the difference of the pseudo-potentials of the wake between the ionization position and the end of the wake bucket is reduced. When this difference is reduced below a certain value, the injection condition breaks leading to termination of the electron injection. Therefore the STII conditions differ from previous ionization injection experiments in the following two major points; the first is the use of unmatched laser spot size in order to allow for a significant evolution of the laser pulse (recalling the so-called matched condition 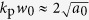, where *k*_p_ is the plasma wave number and *w*_0_ is the laser spot size). The second is the use of low high-Z gas concentrations, typically less than one percent. A recent proof-of-principle experiment^26^ reported an enhancement in the electron acceleration, most likely due to the STII. However so far, there has been no detailed investigation on this mechanism, including its flexibility toward changes in laser-plasma parameters, and in particular its applicability for long laser-plasma acceleration lengths which are needed for accelerating electron beams to GeV energies.

In this paper, we report experimental results to demonstrate the robustness and scalability of the self-truncated ionization injection scheme. We observed its effectiveness and scalability with both the laser power (>100 TW) and the interaction length (1 cm), which led to the generation of 1.2 GeV electron beam with a preserved energy-spread of a few percent. Compared with the results from self-injection and earlier ionization injection experiments, the STII scheme makes the generation of narrow-energy spread GeV electron beams from the LWFA more simple and controllable.

The reported experiments were conducted using a compact state-of-the art Ti: sapphire laser system at the Key Laboratory for Laser Plasmas of Shanghai Jiao Tong University. A schematic diagram of the experimental setup is shown in Fig. 1, more information are given in Methods section. In the following, the results are divided into two parts; in the first part we present results from 30 TW-level laser pulses while the results from above 100 TW-level laser pulses are presented in the second part.

## Results and Discussions

### Results obtained by 30 TW laser pulses

Before presenting the results obtained with He-N_2_ gas mixture, we first recall our results obtained by self-injection in pure He gas jet under unmatched laser-plasma conditions. At low plasma densities^27^ we observed energy spectra of multiple electron bunches having QME features up to 120 MeV with a large energy-spread up to >50%. Simulations revealed that the self-injection process was initiated during evolution of the LWFA from weakly nonlinear into the nonlinear bubble regime. Such evolution occurred when the laser *a*_0_ was enhanced from 1.2 to 3.2 due to a strong self-focusing of the laser pulse. At high plasma densities (*n*_e_ ≈ 1.3 × 10^19^ cm^3^) the interaction became unstable such that the laser beam was broken into several filaments and consequently the electron beam was also broken into multiple beamlets. Therefore, it is concluded that the SI in pure He at *unmatched* laser-plasma parameters is not the proper injection mechanism to achieve high-quality electron beams (means beams having a percent-level energy-spread).

In the present experiment, Fig. 2 shows a series of electron energy spectra obtained using 30 TW-level laser pulses interacting with 4-mm-long He-N_2_ gas mixture of various nitrogen concentrations in the range 0.1%–1%. The laser-plasma parameters *k*_*p*_*w*_0_ and 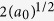 are unmatched and in the ranges ≈10.8–12.2 and ≈2.0–2.1, respectively. Figure 2a shows electron spectra generated from a mixture of 99.9% He gas with 0.1% N_2_ gas. One can notice that in this case the resulting QME beam energy reached ≥200 MeV (~2 times higher than the SI results^27^) and the energy-spread has been reduced to the level of 10% (Fig. 2d) (smaller than the beams from the SI). At such very-low N_2_ gas concentration, there is a self-injection (from the majority 99.9% helium electrons) along with the ionization injection from the nitrogen’s inner-shell electrons; both injection mechanisms contribute to the total electron trapping. By increasing the N_2_ gas concentration to 0.5% we noticed significant reduction of the beam energy-spread to a few percent and the enhancement (Fig. 2b) of the electron energy. Figure 2e is the deconvoluted energy spectrum (of the result in Fig. 2b, right panel) for 412 ± 10 MeV, 80 pC electron beam with an energy spread of ≈5% (FWHM) and beam divergence of 7.1 mrad. In this case, the ionization injection from the nitrogen gas appears to dominate the electron trapping process. By further increasing the N_2_ gas concentration to 1%, a decline in the electron beam parameters (lower energy and larger energy spread) has been observed (Fig. 2c,d). This means that a nitrogen concentration of 1% is relatively high to produce high-quality beams. Therefore, the optimal nitrogen gas concentration at our experimental conditions is around 0.5%. By increasing the N_2_ concentration furthermore, we expect a scenario similar to previous experiments^28^,^29^ which used pure N_2_ gas where electron beams of lower energies (~130 MeV) and larger energy-spreads (~40%) were observed. Figure 2d summarizes the QME electron energy from the He-N_2_ gas mixture at various N_2_ concentrations from 0% (pure He) to 100% (pure N_2_). The error bars represent the standard deviation to the mean values and each point is an average of 7 typical spectra.

Several conclusions can be drawn from Fig. 2. *First*, for the He + N_2_ mixture with N_2_ concentrations of ≤1%, the electron beam parameters (particularly the energy and energy-spread) are enhanced if compared with the beams obtained from pure He by self-injection or previous experiments using 5–100% of nitrogen by the ionization injection. *Second*, there is a general trend for getting high-quality beams through an optimal nitrogen (doped gas) amount which determines the dominance of the ionization injection over the self-injection in this regime. It is found that by changing the N_2_ concentration, one can control the electron beam peak energy and energy spread; this is a key result of this paper. *Third*, through many electron spectra (see Fig. a–e and Tables1–5 in the Supplementary Materials section) we observed the robustness of the current scheme of ionization injection in obtaining multi-hundred MeV, tens pC electron beams with a narrow energy-spread using 30 TW-level laser pulses. The scaling formulas introduced in ref. 30, which assumes the satisfaction of the matching condition 
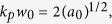
 might not be suitable in predicting the observed results. This is because in the matched case there is almost no evolution of the laser pulse spot size, whereas our scheme mainly relies on the evolution of the laser pulse (as will be shown below). Therefore, we calculate the dephasing length^1^ from the typical relation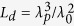, which gives >5 mm (for n_e_ = 5 × 10^18^ cm^−3^, the used density for the He + N_2_ gas mixture 0.1 to 1% nitrogen concentration). Since the gas length was only 4 mm, we can exclude the role of dephasing and confirm the role of ionization injection in the formation of the observed electron beams in Fig. 2.

We have conducted 3D−PIC simulations using the code OSIRIS^31^ for the unmatched conditions close to those used in the experiment (see Methods). The evolutions of the normalized maximum laser electric field E_max_(proportional to square root of the laser intensity; E_max_ is in unites of 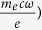 and the wake wave pseudo-potential difference between the internal electron ionization position and the end of the first wake wave are both shown in Fig. 3a. The figure shows that during the first one millimeter of interaction the laser intensity was below the ionization threshold for the nitrogen’s inner-shell (which requires *E*_*max*_ > 1.9). Then, during the remaining ≈3 mm of interaction length the laser intensity evolved to values higher than the ionization threshold of the internal electrons (N^5+^, N^6+^) which are used for the ionization injection. However, the injection happened only within a very limited region in space which is determined by both the ionization and the wake wave pseudo-potential difference (Δ*ψ*)^25^. The ionized K-shell electrons can be trapped only if they gain enough energy from the wakefield (the threshold is Δ*ψ* > 0.9, the dashed blue line in Fig. 3a) before they slip away from the back of the wake wave. In Fig. 3a we can see that there are two regions where the ionization injection of electrons has occurred (1000 μm ≤ *z* ≤ 1200 μm and 2800 μm ≤ *z* ≤ 2900 μm) and the injection is truncated elsewhere. Correspondingly, the injected charge evolution is shown in Fig. 3b. In the first (main) injection about 100 pC of charge is injected into the wake wave. The second (minor) injection gives only 25 pC charge which has relatively low-energy. A snapshot for the laser pulse and electron beam is shown in Fig. 3c, as one can see, almost purely monoenergetic high-energy electrons are accelerated close to the bucket’s center while the low-energy electrons left behind are easily lost due to the laser and wake wave evolution. Fig. 3d shows the energy spectrum of the narrow energy-spread 380 MeV electron bunch which characterizes the STII. Fig. 3c clearly shows the interaction between the laser pulse and the accelerated electron bunch. Such interaction can have two effects; the first one is the enhanced acceleration of the electron bunch due to the direct laser acceleration^32^ (DLA) mechanism, this effect can help explaining the enhanced beam energy (>400 MeV) from the STII as compared with the SI case (120 MeV). The second effect is the increase in the transverse momenta of the accelerated electrons which leads to increase the spatial beam size. This effect can be seen in Fig. 2a–c, where the divergence of the electron beam from the STII is ≈7–9 mrad, which is roughly twice the typical divergence angle for the beams produced by the self-injection^15^.The 3D-PIC simulation presented in Fig. 3 confirms the essential features of the self-truncated ionization injection phenomenon.

### Results obtained by 120 TW laser pulses

To demonstrate the applicability of the STII mechanism for GeV electron beam acceleration in LWFA, we increased the laser power to ≥100 TW. Figure 4a,b show electron energy spectra generated by ≥100 TW laser pulses interacting with 4 mm gas jets of pure He and a mixture of 0.5% N_2_ and 99.5% He, respectively, at the plasma density of 6.5 (±0.5) × 10^18^ cm^−3^. Figure 4e is the deconvoluted energy spectrum of the electron beam shown in Fig. 4b. The beam parameters by ionization injection (*E*_*QME*_ = 530 ± 8 MeV, Q (charge) = 25 pC, Δ*E*/*E* ≈ 8%, divergence angle of 5.2 mrad) in Fig. 4b are higher quality than the beam produced by the self-injection (*E* = 300 ± 4.5 MeV, Q = 21 pC Δ*E*/*E* ≈ 25% divergence angle of 7.6 mrad) in Fig. 4a, for unmatched laser-plasma parameters. However, there is a small tail-bunch of electrons at a lower energy in Fig. 4b, which might be the secondary-injected bunch that was mentioned in the above 3D simulations, or due to the fact that both the plasma density and laser power were too high, therefore the self-injection from the pure He also occurred along with the ionization injection. In either case, the early onset of ionization injection due to its lower threshold, considerably dominated the process and suppressed the self-injection^33^.

In order to achieve GeV electron beam energy, we replaced the 4 mm gas jet with a 1 cm gas jet operating at low density to overcome the dephasing issue. Figure 4c,d show the electron spectra produced from ≥100 TW laser pulses interacting with 1 cm gas jets of pure He and a mixture of 0.3% N_2_ and 99.7% He, respectively. The plasma density was 1.8 × 10^18^ cm^−3^ and the laser-plasma parameters are unmatched: 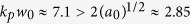. Figure 4c (self-injection) shows multiple QME electron bunches at 150 MeV, 400 MeV, and 800 MeV; the total charge of those bunches altogether is 12 pC. Similar results were observed in pervious self-injection experiments^34^,^35^,^36^,^37^ at matched laser -plasma parameters. In contrast to the electron spectrum from the self-injection, an electron beam with a monoenergetic peak at ≈1.2 ± 0.03 GeV, an energy-spread (FWHM) of ≈7%, total charge of 16 pC and divergence angle of 4.7 mrad is observed by ionization injection from the gas mixture (Fig. 4d,f). As we observed in the 30 TW-level laser case, the electron beam parameters are dramatically enhanced due to the ionization injection. This shows the advantage of the STII over the self-injection for the unmatched laser-plasma parameters. This is the first result (to the best of our knowledge) on single-stage LWFA using ionization-injection to demonstrate monenergetic electron beams beyond 1 GeV.

To better understand the physics of the acceleration over 1 cm length, we have conducted 2D-PIC simulations using OSIRIS and the simulation results are shown in Fig. 5. In this case the initial laser intensity was high-enough to ionize the inner nitrogen K-shell. However, (similar to the 30 TW-level laser case), the ionization-injection condition is satisfied only in limited locations indicated by the values of Δ*ψ* curve above the Δ*ψ*_*th*_ dashed line, as shown in Fig. 5a,b. The detailed electron injection positions are shown in the longitudinal phase-space graph in Fig. 5c. There are two injection locations, the main injection occurred during the first 1 mm where a large group of electrons was trapped then a rapid truncation took place. The truncation continued between *z* ≈ 1 mm and *z* ≈ 4 mm, as a result, the main injection forms a 4% energy-spread GeV-level electron beam at last, as shown in Fig. 5d. The secondary injection happened from *z* = 4 mm to 6 mm where electrons were injected almost continuously; those electrons composed the lower energy 19% energy-spread electron bunch. As one can see, the physical scenario for the ≥100 TW and 30 TW cases is identical; therefore we can conclude that the self-truncated ionization injection mechanism is applicable to cm-scale single-stage LWFA at high laser power and leads to narrow energy-spread GeV electron beams. Future experimental and theoretical work should investigate the applicability of the STII mechanism for longer lengths using the currently available petawatt-class lasers, where one can expect significant enhancement in the beam quality at the level of 5–10 GeV electron energy.

In conclusion, using 30–120 TW-class laser pulses we experimentally demonstrated monoenergetic electron beams from single-stage LWFA with peak energy up to 1.2 GeV by the self-truncated ionization injection (STII) for the first time. The experiment has been carried out in unmatched laser-plasma parameter regime, where the (STII) mechanism works, leading to electron beams with narrow energy-spread (5% at 412 MeV and 7% at 1.2 GeV) in contrast to the broad or continuous spectra (25–100%) produced by the usual ionization-injection that employs matched laser-plasma parameters, (see Table 5 in the Supplementary materials for detailed comparison with previous results). The STII is robust and scalable, as well as more controllable (for example, through the nitrogen concentration in the mixture) compared with the self-injection mechanism. All these advantages are very important for practical applications of laser-plasma electron beams. Of particular interest, is the use of high-quality electron beams to seed next generation light sources such as table-top X-ray free electron laser (XFEL). A recent^4^ GENESIS simulation study have shown a FEL gain by the injection (seeding) of a decompressed multi-hundred MeV electron beam having a percent-level energy-spread from a laser-plasma accelerator into an optimized undulator in which the Pierce parameter and gain length were properly balanced^4^. Our observation of narrow energy-spread electron beams by the STII mechanism is a step forward toward the realization of table-top XFEL.

## Methods

### Laser and targets

**Figure 1:**
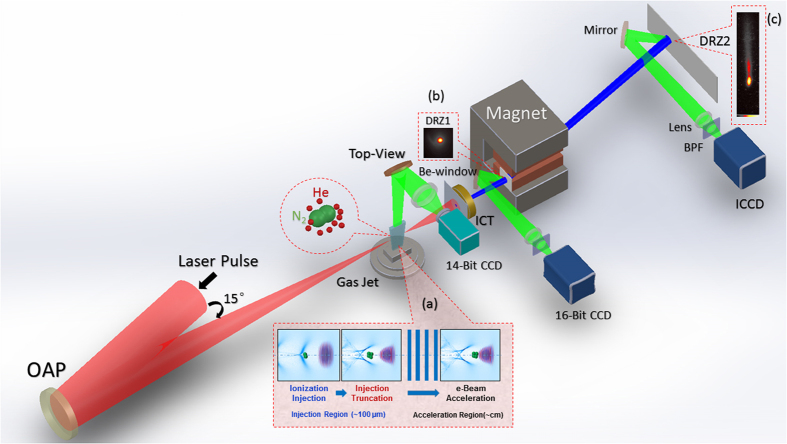
Schematic diagram of the experimental setup. Up to 118 TW 30 fs laser pulses are focused down to 28 μm spot size with an OAP (f = 2 m) onto 4 mm or 1 cm supersonic gas jet of He and N_2_ gas mixture. The self-truncated ionization injection (STII) mechanism is illustrated in inset (**a**). Inset (**b**) shows a fixed fluorescent DRZ screen for monitoring the electron beam pointing and divergence angles before entering the magnet. Inset (**c**) is an electron beam energy spectrum. Top-view imaging system monitors the laser-plasma. ICT stands for integrating-current transformer used to measure the beam charge. The laser-produced plasma density was probed (not shown) in earlier experiments by the authors via interferometry using a 100 fs probe beam and by FRS diagnostic.

**Figure 2:**
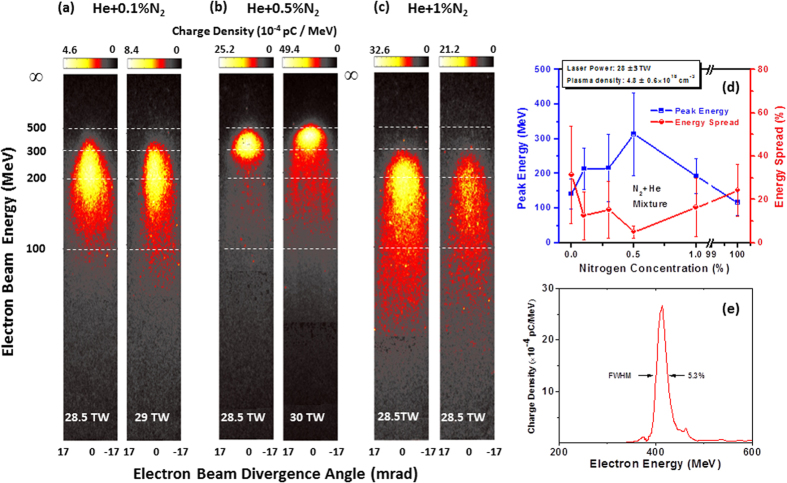
Electron beam energy spectra vs varying nitrogen gas concentration. (**a**–**c**) Electron energy spectra versus nitrogen concentrations (0.1% to 1%) for ≈30 TW laser power and 5 × 10^18^ cm^−3^ plasma density. The laser-plasma parameter *k*_p_*w*_0_ = 11.9 unmatched with 2(a_0_)^1/2^ = 2 (**d**) peak (not the maximum) energy and energy-spread (FWHM) of the generated electron beams. The laser-plasma parameters are *k*_p_*w*_0_ ~ 10.8–12.2, unmatched with 2(*a*_0_)^1/2^ ≈ 2–2.1. Zero percent nitrogen concentration means pure He gas, while 100% means pure nitrogen gas. Each data point is the average of 7 typical spectra and the error bars illustrate 2σ, where σ is the standard deviation of the electron spectra and energy spread from the mean values. (**e**) Deconvoluted electron spectrum of the right panel image in (**b**) for 412 MeV beam.

**Figure 3:**
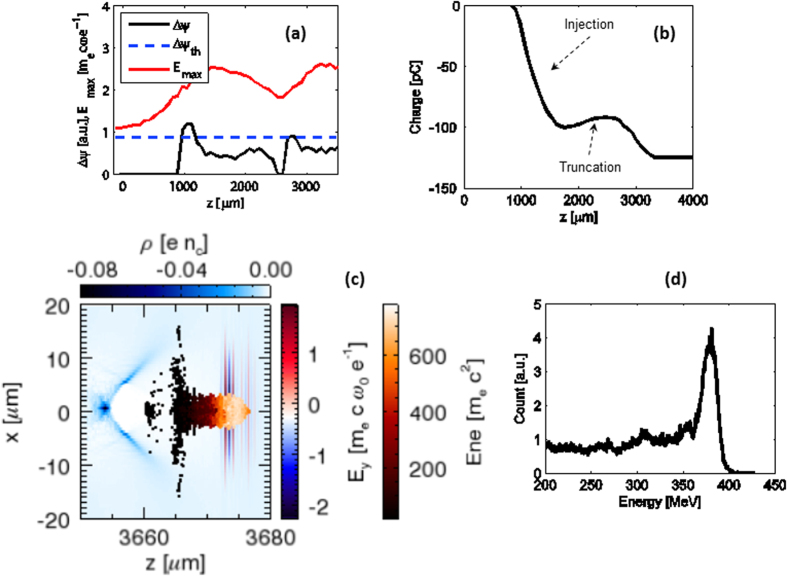
3D-PIC simulations of the STII for 30 TW-level laser pulse. (**a**) Evolution of the maximum laser electric field and pseudo-potential difference. (**b**) Injected electron charge along the propagation direction. (**c**) Snapshot of laser pulse and electron beam after propagating 3.6 mm in the plasma. (**d**) Energy spectrum of the accelerated electrons.

**Figure 4:**
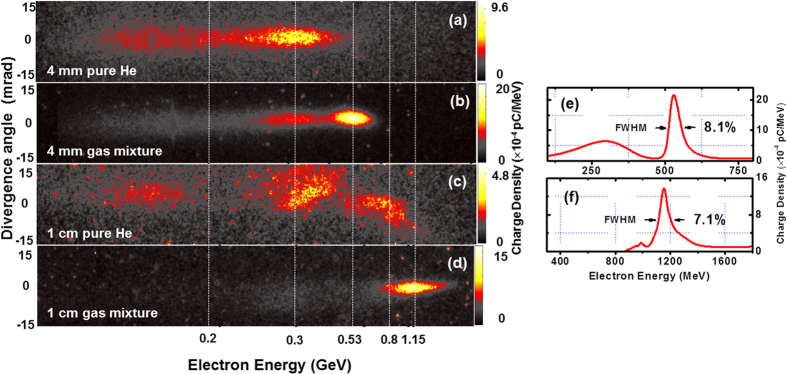
Electron energy spectra from 120 TW-level laser pulses. Spectra obtained from 4 mm gas jet of (**a**) pure He and (**b**) 99.5% He and 0.5% N_2_ gas mixture. Spectra obtained from 1 cm gas jet of (**c**) pure He and (**d**) 99.7% He and 0.3% N_2_ gas mixture. (**e**) and (**f**) are deconvoluted energy spectra of the images in (**b**,**d**), respectively. The laser-plasma parameters are given in the text.

**Figure 5:**
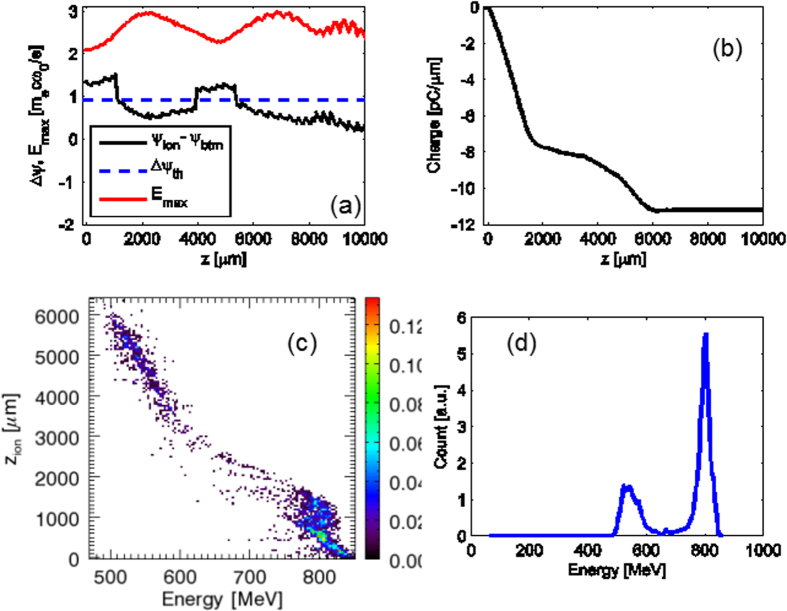
2D-PIC simulations of the STII for 120 TW-level laser pulse. (**a**) Evolution of the maximum laser electric field and wake pseudo-potential difference. (**b**) Injected beam charge along the propagation. (**c**) Longitudinal phase-space showing the injection locations of the electrons. (**d**) Energy spectrum of the accelerated electrons after 1 cm laser propagation in the gas mixture, the main bunch due to STII is GeV-level with 4% (FWHM) energy spread.

Figure 1 shows a schematic of the experimental setup. Up to 118 TW 30 fs, 800 nm laser pulses are focused by *f*/20 off-axis parabolic mirror (OAP) onto 4 mm or 1 cm-long slit-shaped supersonic gas jet, which ejects a mixture of He and N_2_ gases. The 1/*e*^2^ radius of the Gaussian intensity distribution of the laser focus spot was 28 μm, the Strehl ratio of the focal spot was 0.5 and the Rayleigh length *Z*_*r*_ is was 3.1 mm. For 118 (30) TW pulses, the focused peak intensity and the corresponding normalized vector potential *a*_0_ were 8.7 × 10^18^ (2.6 × 10^18^) W/cm^2^ and 2.1 (1.1), respectively. The density of the laser-produced plasma from both nozzles (4 mm and 1 cm) was probed by interferometery and forward Raman scattering (FRS) diagnostics in previous experiments and comparted with hydrodynamic calculations of the gas density^38^,^39^,^40^. The nozzle was positioned below the laser spot at a vertical height of 2 mm and the gas valve was triggered 4.5 ms (which is the time it takes for the gas density to reach maximum at nozzle exit) before arrival of the laser pulse. By varying the backing pressure of the gas jet we could change the plasma density from 10^18^ cm^−3^ to 10^19^ cm^−3^. In order to achieve accurate He + N_2_ gas mixing ratio, commercially available bottles with industrial standards (mixing error of 0.01%) were used.

### Electron beam detection

Two fluorescent DRZ screens were used to monitor the electron beams. The DRZ1 which was imaged into a 16-bit CCD was placed (fixed) before a 6 cm-long dipole magnet (0.9 T) to monitor the pointing angle of the electron beams. The DRZ2 was imaged into an ICCD and placed after the magnet to diagnose the energy spectrum. The distances from the gas jet to DRZ1, magnet, and DRZ2 were 72, 81, and 161 cm, respectively. A particle trajectory code was used to calculate the electron energy shot-to-shot taking into account the beam pointing angle (at DRZ1) before the magnet and the code was written based on the method adapted from ref. 41. To get the electron beam energy-spread, a computer code was written to deconvlove the spectrum after the magnet from the beam size for each shot. With the 81-cm distance between the magnet entrance to DRZ2, the electron energy resolutions for the 30 TW-level laser results were: 2.1% at 304 MeV and 2.8% at 570 MeV. In order to measure GeV electron energies, the small magnet was replaced with a 16 cm-long dipole-magnet having a field intensity of 1 Tesla, and the energy resolutions at 735 MeV and 1.4 GeV were 1.6% and 2.4%, respectively. An integrating current transformer (ICT) coupled with a beam charge monitor (BCM) unit was used for measuring the beam charge. The electron beam was coupled to the detection system (which was installed outside vacuum) via a 100 μm-thick beryllium window.

### PIC simulations

In the 3D (2D) simulation for low (high)-power, 800 nm laser pulse with normalized vector potential *a*_0_ = 1.11 (2.11) and 30 fs duration was focused at the beginning of the plateau part of a gas-plasma mixture. The laser power was ~27 (118) TW and the laser-plasma was unmatched 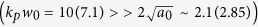. To reduce the simulation time, we have not included the neutral He, instead we included a background pre-ionized plasma and nitrogen gas. The plasma density was *n*_e_ = 4.3 × 10^18^ (1.8 × 10^18^) cm^−3^ and the N_2_ atomic number density was *n*_N_ = 3.39 × 10^16^ (4.4 × 10^15^) cm^−3^, therefore the ratio 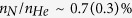. The gas mixture was uniform except for 200 μm up-ramp vacuum-plasma transition layer in front of the plateau plasma. The total simulation box size was 40 × 200 × 200 μm^3^ (100 × 180 μm^2^) with the grid number of 1280 × 200 × 200 (3072 × 256) and the simulation time step was 0.104 (0.108) fs. In the simulation, 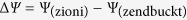 is the potential difference between the electron ionization position and the end of the first wake wave. The threshold for ionization injection is given by 
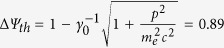
 (0.93) for the low power (high power), where the normalized transverse momentum is estimated to be the normalized laser vector potential at ionization position *p* = 1.97 (1.9) and the Lorentz factor of the wake phase velocity is estimated by the linear theory *γ*_0_ = *ω*/*ω*_p_ = 20.1 (31).

## Additional Information

**How to cite this article**: Mirzaie, M. *et al.* Demonstration of self-truncated ionization injection for GeV electron beams. *Sci. Rep.* **5**, 14659; doi: 10.1038/srep14659 (2015).

## Supplementary Material

Supplementary Information
