# Supplementary material for: Demonstration of self-truncated ionization injection for GeV electron beams
Source: Sci Rep. 2015 Oct 1;5:14659. doi: 10.1038/srep14659 (PMC4589762; doi:10.1038/srep14659)
Supplement: Supplementary Information [file srep14659-s1.pdf]

Supplementary Materials For

Demonstration of self-truncated ionization injection for GeV electron beams

M. Mirzaie[1, 2,*], S. Li[1, 2,*], M. Zeng[1,2], N. A. M. Hafz[1, 2], M. Chen[1, 2], G. Y. Li[1, 2], Q. J. Zhu[2], H. Liao[2],

T. Sokollik[1, 2], F. Liu[1, 2], Y. Y. Ma[2, 3], L.M. Chen[2, 4], Z. M. Sheng[1, 2, 5], J. Zhang[1, 2]

[1] Key Laboratory for Laser Plasmas (MOE) and Department of Physics and Astronomy, Shanghai Jiao Tong University, Shanghai 200240, China. [2] IFSA Collaborative Innovation Center, Shanghai Jiao Tong University, Shanghai 200240, China [3] College of Science, National University of Defense Technology, Changsha 410073, China. [4] Beijing National Laboratory of Condensed Matter Physics, Institute of Physics, CAS, Beijing 100190, China. [5] SUPA, Department of Physics, University of Strathclyde, Glasgow G4 0NG, UK. [*]These authors contributed equally to this work. Correspondence and requests for materials should be addressed to N.A.M.H. (email: nasr@sjtu.edu.cn), or M.C. (email: minchen@sjtu.edu.cn), or J. Z. (email: jzhang1@sjtu.edu.cn).

30 TW-level laser shots (Our Results)

A series of electron energy spectra generated by 30 TW-level laser-driven 4 mm nozzle of pure He (self-injection):

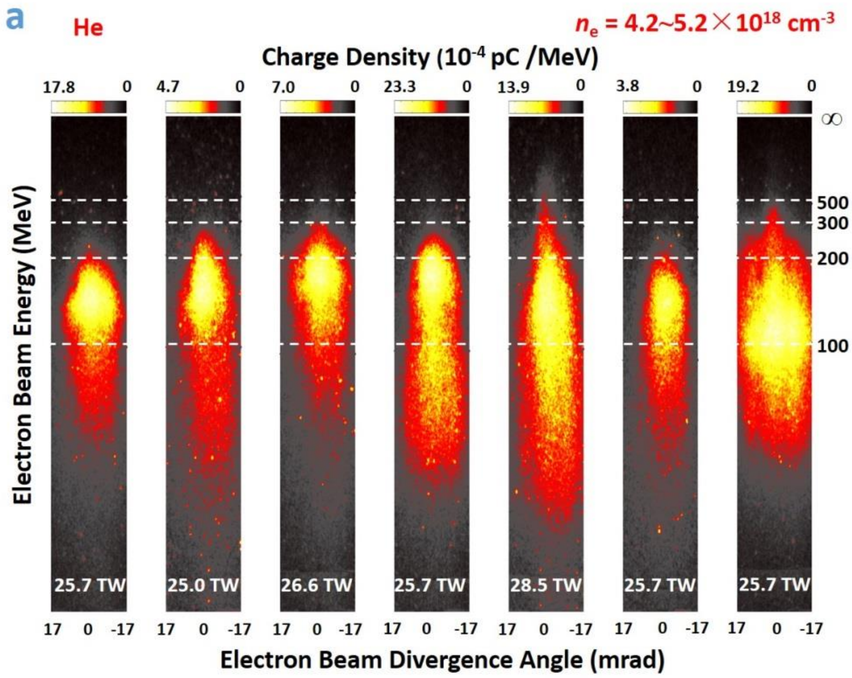

Fig. (a)

A series of electron energy spectra generated by 30 TW-level laser-driven 4 mm nozzle of helium - nitrogen gas mixtures for different nitrogen concentrations as shown on each set below:

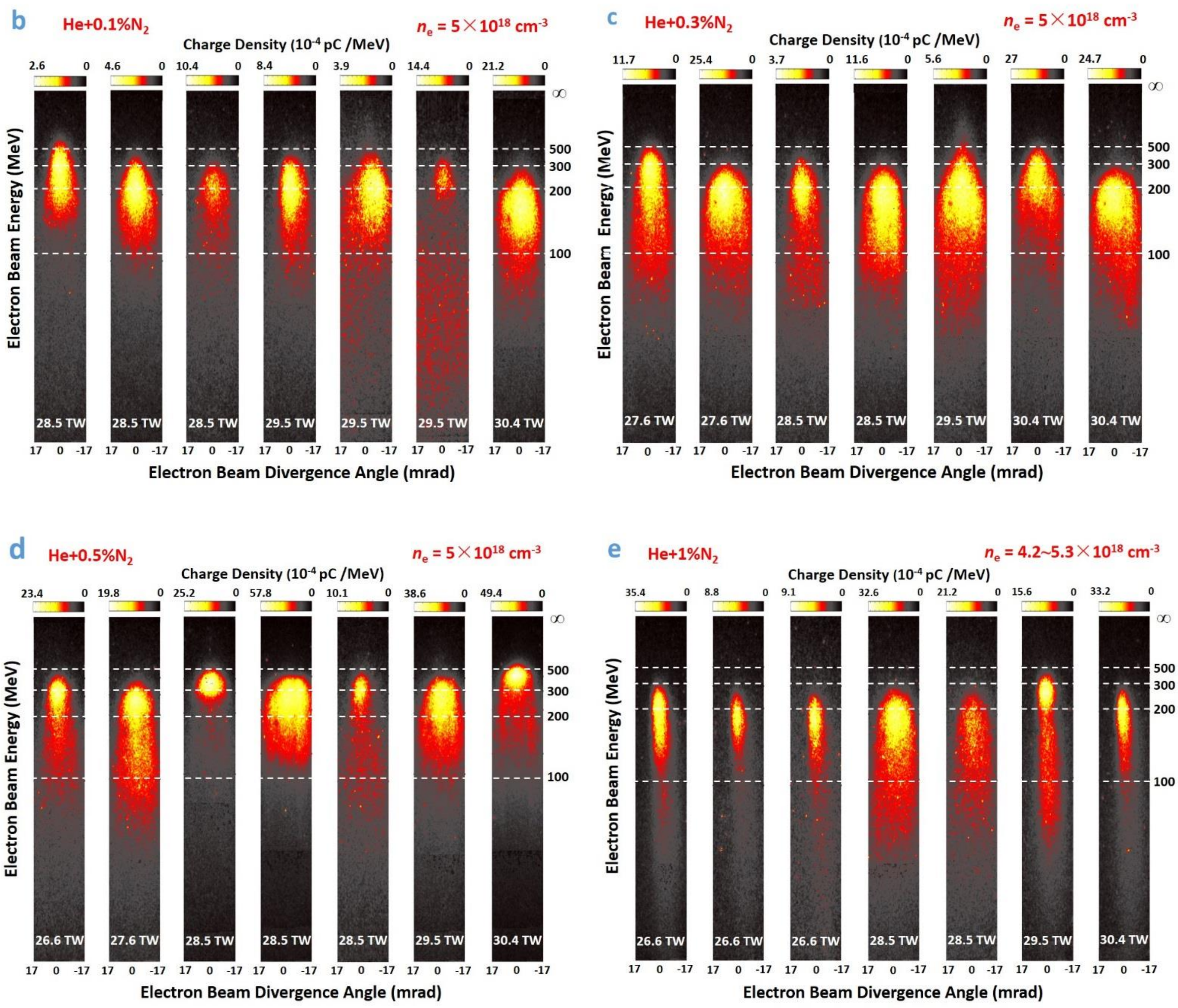

Fig. (b, c,d,e)

Table 1. Peak Energy of 7 Typical Electron Beam Spectra in Figs. a–e

Nitrogen Concentration (%)	Peak Energy (MeV)							Average of Peak Energy (MeV)	1σ Standard Deviation (MeV)	2σ Standard Deviation (MeV)
0 (pure He)	139	151	161	166	137	132	102	141	21.4	42.8
0.1	256	211	200	210	242	213	164	213	29.6	59.2
0.3	283	175	201	183	206	287	176	215	48.7	97.4
0.5	300	252	370	292	321	251	412	314	59.6	119.2
1	200	182	180	184	161	241	199	192	25.1	50.2

Table 2. Corresponding Energy Spread of 7 Typical Electron Beam Spectra in Figs. a–e

Nitrogen Concentration (%)	Corresponding Energy Spread (%)							Average of Energy Spread (%)	1σ Standard Deviation (%)	2σ Standard Deviation (%)
0 (pure He)	23	27.9	20	27	52.6	39.7	29.2	31.3	11.2	22.4
0.1	21.3	15.9	7.5	17.3	9.4	9	7.4	12.5	5.6	11.2
0.3	23.2	5.3	18.3	10.5	21.4	18.3	10.7	15.4	6.6	13.2
0.5	5	3.9	5.9	2.6	5.4	7	5.3	5.0	1.4	2.8
1	24	19	21.4	7.9	10.8	9.2	21.8	16.3	6.8	13.6

Table 3. Corresponding Beam Charge of 7 Typical Electron Beam Spectra in Figs. a–e

Nitrogen Concentration (%)	Corresponding Beam Charge (pC)							Average of Beam Charge (pC)	1σ Standard Deviation (pC)	2σ Standard Deviation (pC)
0 (pure He)	87	14	30	77	46	10	102	52.3	36.7	73.4
0.1	5	9.2	20.2	17.3	35.3	10.1	48.4	20.7	15.7	31.4
0.3	26.2	58.6	8.1	26.4	15.1	54.5	58.5	35.3	21.4	42.8
0.5	47.4	45.4	37.8	109	20.2	78.7	79.8	59.8	30.5	61
1	52.5	13.1	18.2	79.8	46.2	27	47.4	40.6	23.1	46.2

Table 4. Corresponding Beam Divergence of 7 Typical Electron Beam Spectra in Figs. a–e

Nitrogen Concentration (%)	Corresponding Beam Divergence (mrad)							Average of Beam Divergence (mrad)	1σ Standard Deviation (mrad)	2σ Standard Deviation (mrad)
0 (pure He)	14.5	17.5	17.7	9	13.8	19.1	15.6	15.3	3.4	6.8
0.1	7.8	7.8	7.6	6.4	18.2	7.6	11.3	9.5	4.1	8.2
0.3	8	9.7	8.3	8.5	9.2	9.4	9.9	9	0.7	1.4
0.5	7.4	9	7.4	9	7.1	10.6	7.1	8.2	1.3	2.6
1	6.9	6.7	6.7	8.7	7.6	6.9	7.8	7.3	0.7	1.4

Table: 5 Brief summary and comparison with relevant results

Author/ Group	Journal /Year	Injection Method	Laser Power & Pulse Duration	Electron Beam Energy Spectrum	Energy spread	Charge & Divergence	Gas Type	Target design	No. of Stages
C. Rechatin et al., LOA	PRL (2009)	Optical Injection	30 TW 33 fs	170 MeV single bunch	3.1 % (rms)	10 pC & 4.5 mrad	He	3 mm gas jet	2 colliding laser pulses
S. Kneip et al., IC	PRL 2009	Self-injection	200 TW 55 fs	200, 300, 800 MeV multi-bunches	25 %	90 pC & 3.6 mrad	He	10 mm gas jet	Single stage
C. Clayton et al., UCLA	PRL 2010	Ionization Injection	110TW 60 fs	1.45 GeV continuous	100 %	3.8 pC & 4.4 mrad	97 % He + 3% CO_2	13mm gas cell	Single stage
B. Pollock et al., UCLA	PRL 2011	Ionization Injection	60 TW 60 fs	460 MeV Single bunch	5 %	35 pC & 2.3 mrad	(99.5 % He + 0.5 % N_2) & He	10 mm gas cell	Two stages
J.S. Liu et al., SIOM	PRL 2011	Ionization Injection	43TW 40 fs	800 MeV multi-bunches	25 %	3.7pC & 2.6 mrad	(94 % He + 6 % O_2) & He	4 mm gas cell	Two stages
A. Gonsalves et al., LBNL	Nature Physics (2011)	Density down-ramp	40 TW 40 fs	340 MeV single bunch	11 %	10 pC & 2.5 mrad	He+H	33 mm capillary discharge	Two stages
Our work (1) SJTU	2015	Self-truncated Ionization Injection	30 TW	412 MeV single bunch	5 %	80 pC & 7.1 mrad	He 99.5 % +N_2 0.5 %	4 mm gas jet	Single stage
Our work (2) SJTU	2015	Self-truncated Ionization Injection	118 TW	1.2 GeV single bunch	7 %	16 pC & 4.7 mrad	He 99.7 % +N_2 0.3 %	10 mm gas jet	Single stage
